# Older Breast Cancer Survivors’ Cognitive Response to Qigong/Tai Chi Easy: An Exploratory Analysis

**DOI:** 10.1093/geroni/igab046.2581

**Published:** 2021-12-17

**Authors:** Dara James, Linda Larkey, SeungYong Han

**Affiliations:** Arizona State University, Phoenix, Arizona, United States

## Abstract

Increasing rates of breast cancer coupled with improvements in treatment means the number of breast cancer survivors (BCSs) is growing. BCSs frequently report persistent cognitive deficits (i.e., “cancer-related cognitive impairment”) that impacts QOL and treatment compliance. Older (≥65 years old) BCSs are more likely to experience cognitive decline and impairment, partly due to the biological process of senescence. In the context of a larger RCT of BCSs (ages 45-75; stages 0-III), we evaluated cognitive function/performance effects on among the older participants (ages 65-75) of 8-weeks Qigong/Tai Chi Easy (QG/TCE) compared to education control (EdC). Cognitive function was measured using the Functional Assessment of Cancer Therapy-Cognitive Function (FACT-COG), including: perceived cognitive impairment (PCI), and perceptions of effects of cognitive function on quality of life (PCQOL). Cognitive performance was measured using the Wechsler Adult Intelligence Scale-Third Edition (WAIS-III): Digit Span (DS) and Letter-Number Sequencing (LNS). A multilevel model with random intercept was used to examine GroupXTime interactions: The majority of participants (N= 32) (M age= 69.7) were white (84%). Changes in WAIS-III DS, LNS and FACT-COG PCI were not statistically significant, but effect sizes were small to medium. The interaction between group and time was significant for FACT-COG PCQOL (p= 0.033) with a medium effect size, 0.14. Findings from this exploratory analysis of the larger study suggests that older BCSs’ participation in QG/TCE may improve perceptions of effects of cognitive function on quality of life. Such improvements may increase cognitive-related self-efficacy, overall QOL and treatment compliance among older BCSs.

